# Severe Enterovirus Infections in Infants <3 Months of Age and the Importance of Medical History

**DOI:** 10.34763/jmotherandchild.20202403.2022.d-20-00007

**Published:** 2021-01-29

**Authors:** Anna Olchawa-Czech, Katarzyna Ptak, Izabela Szymońska, Przemko Kwinta

**Affiliations:** 1 Faculty of Medicine, Institute of Paediatrics, Jagiellonian University Medical College, Cracow, Poland

**Keywords:** enteroviruses, viral sepsis, newborns, infants 3 months old

## Abstract

**Background:**

Enteroviral infections in infants <3 months of age are frequent and under-diagnosed even though they can be life-threatening. Properly conducted subjective examination, which is repeatedly neglected, plays a key role in the diagnosis and treatment of these infections.

**Materials and methods:**

Analyses included children <3 months of age with confirmed enterovirus infection, hospitalised in the Department of Paediatrics from January 2019 to February 2020. Infections were confirmed by reverse transcription polymerase chain reaction in the cerebrospinal fluid using Neuro9 FTD set and in the stool using PB-03/Neuro; antibodies were determined in one patient.

**Results:**

This study presents a detailed description of three cases with confirmed enterovirus infection and a positive epidemiological history. The cases involve viral sepsis, myocarditis with arrhythmia and circulatory failure, and meningitis with seizures. In addition, the details of 10 patients hospitalised in the Children’s Clinic with a confirmed enterovirus infection are presented. Based on these cases, a significant influence of family history-taking on the diagnosis and implementation of appropriate treatment was found.

**Conclusion:**

In most of the analysed cases, family history of viral infection was positive. In patients with the most severe course of the enterovirus infection, accurate epidemiological history is extremely important, and the suspicion of viral infection and securing appropriate materials for testing may significantly speed up the diagnosis in the newborn and help to implement an appropriate treatment.

## Introduction

Enterovirus infections are one of the common causes of hospitalisation in paediatric wards. The course of these infections can vary from slight infections of the gastrointestinal tract or upper respiratory tract to severe infections of the central nervous system and myocardium. Although enterovirus infections are common in the paediatric population, a proper diagnosis, especially in the youngest patients, is difficult to make. This is caused by lack of characteristic symptoms that can be attributed to this group of diseases only.

Mild infections are generally classified as unspecified viral infections. Severe cases are initially suspected to be serious bacterial infections, such as sepsis or meningitis, and treated aggressively. Meanwhile, in many cases, a carefully collected family history that includes parents, siblings and other co-residents could make enterovirus infections an important

point of differential diagnosis, resulting in the collection of microbiological tests and implementation of appropriate treatment.

The aim of this article is to highlight the key role of history-taking in the process of differential diagnosis of infections in newborns and young infants.

## Materials and methods

This paper presents the characteristics of patients <3 months of age with confirmed enterovirus infection hospitalised in the Department of Paediatrics from January 2019 to February 2020. Out of the 10 described cases, seven were diagnosed with meningitis, two with myocarditis and one with viral sepsis. These infections were confirmed in three patients using FTD Neuro 9 kit in CSF and in the stool of five patients using PB-03/Neuro. One patient with myocarditis had antibodies, which were positive in immunoglobulin (Ig )-M and IgG classes for coxsackie B1–B6 viruses, and the presence of enteroviral genetic material was confirmed in the biopsy material of the final patient.

## Results

Detailed descriptions of the cases of severe enterovirus infections are presented in. Table 1, with the characteristics of all the analysed patients.

**Table:** Table 1

No.	Month of admission	Age	Family history	Symptoms on admission	CRP [mg/L]	PCT [ng/mL]	Leucocytes [/uL]	Neutrophils [%]	Lymphocytes [%]	CSF cytosis [cells/	Mononuclear cells in CSF [%]	Multinuclear cells in CSF	Glucose in CSF [mmol/L]	Protein in CSF [g/L]	Lactic acid in CSF [mmol/L]	Where infection has been confirmed	TroponinT [ng/L]	Antibiotics	HSV in CSF	Neuroimaging
1.	Il	0/365	Child's mother has fever	Respiratory failure, prematurity	7.9	0.93	8310	60.1	24.4		Examinations not performed					Faeces, tissue biopsy	Examinations not performed	Ampicillin, amikin, meropenem, vancomycin	Samples not collected	Ultrasound scan - normal before the septic shock
2.	X	1/365	Father had hand, foot and mouth disease about month 1 earlier, mother was vomiting on the day she gave birth	Respiratory and circulatory insufficiency, arrhythmia, ascites	<5	Not performed	8370	64.3	22.1		Examinations not performed					Mother's stool	254.7	Ampicillin, amikin, aciclovir	Samples not collected	MRI-features of (minor) intramuscular bleeding and minor haemorrhagic lesions of the cerebellum and the right lobe. Features of immaturity of the white brain matter. There were no signs of encephalitis, (prematurity 29 hbd)
3.	II	19/365	His father had been feverish a few days earlier, older his brother had infection of the upper respiratory tract	Fever, seizures (tonic seizure, strabismus, pushing out the tongue)	<5	0.12	19770	32.4	51.5	1910	98.9	1.1	2	1.22	1.5	Stoo	50.1	Cephotaxim, ampicillin, aciclovir	Negative	Ultrasound-thalamostriate vasculopathy
**4**.	**VI**	**6/52**	No data	Fever, hyperesthesia, abdominal distension	**11.2**	**0.15**	**9170**	**45.2**	**41.9**	**693**	**53.3**	**46.7**	**3**	**2.02**	**1.4**	Stool	**32.4**	Ceftriaxone, amikacin, aciclovir	Negative	Ultrasound-normal
**5**.	**VII**	**7/52**	Members of the household with symptoms of viral infection	Fever, lack of appetite, apathy	**5.9**	**0.16**	**13000**	**32.4**	**47.8**	**673**	**27.5**	**72.5**	**3**	**0,76**	**1.4**	Stool	Examinations not performed	Cephotaxim, amikacin, vancomycin, ceftriaxone, aciclovir	Negative	MRI-normal
**6**.	**X**	**6/52**	No data	Fever, hyperesthesia, difficulty in Eating	**<5**	**0.16**	**3460**	**26.3**	**43.6**	**2**	**100**	**0**	**3**	**0.52**	**1.4**	CSF	Examinations not performed	No.	Negative	Ultrasound-normal
**7**.	**XI**	**1/12**	Older siblings had upper respiratory tract infections Older sister had	Fever, lack of appetite, apathy	**6,6**	**0.17**	**12290**	**48.3**	**39.3**	**530**	**98.7**	**1.3**	**2**	**1.12**	**1.1**	Stool	Examinations not performed	Ceftriaxone, amikin, fluconazole	No data	Ultrasound-normal
**8**.	**XII**	**1/12**	upper respiratory tract infection	Fever, apathy	**£5**	**0.11**	**11380**	**22.2**	**57.3**	**698**	**94.7**	**5.3**	2	**1.2**	1.7	CSF	Examinations not performed	Ampicillin, amikacin, aciclovir	No data	Ultrasound-normal
**9**.		**7/365**	Father had pharyngitis	Fever, apathy, difficulty in Eating	**<5**	**0.2**	**13910**	**30.4**	**53.9**	**492**	**97.5**	**2.5**	**2**	**1.21**	**1.2**	CSF	Examinations not performed	Aciclovir	Negative	MRI-normal
**10**.	**X**	**7/52**	No data	Fever, apathy, difficulty in Eating	**10.7**	**0.18**	**10390**	**14.9**	**71.9**			Examinations	not performed			Stool	**329.2**	Cefuroxime	Sampies not collected	Ultrasound-normal

CRP, C-reactive protein; PCT, procalcitonin; CSF, cerebrospinal fluid; HSV, herpes simplex virus;; MRI, magnetic resonance imaging.

## Case 1 – enteroviral sepsis

A male neonate was transferred to the Neonatal Intensive Care Unit of the Department of Paediatrics in Cracow, on the day of his birth. The child was delivered at 28 weeks’ gestation to a gravida 3, para 3 mother by Caesarean section due to premature placental detachment, and he had a birth weight of 1200 g. The child was assessed using the Apgar scale at 1/3/5/10 minutes getting 6/4/6/6 points, respectively. He required respiratory support and surfactant supply using the INtubation-SURfactant-Extubation (INSURE method: intubation, surfactant supply and extubation), followed by nasal continuous positive airway pressure (nCPAP). On the day of birth, the mother had had a fever, but influenza infection was ruled out.

During admission, the child was in fair condition – with symptoms of dyspnoea – vital and responsive. In laboratory tests, C-reactive protein (CRP) level was <5 mg/L, and blood morphology was normal. Ultrasound studies of the brain, heart, lungs and abdominal cavity did not reveal significant abnormalities. Empirical antibiotic therapy based on amikacin and ampicillin was instituted and then discontinued after the negative results of bacteriological examinations and low inflammatory markers. The child’s condition remained stable; there was no fever. The mother was still feverish for no apparent reason: bacteriological cultures of blood and urine were negative; inflammation markers were low.

On Day 7, the child’s condition deteriorated rapidly; centralisation of circulation was observed on physical examination. The ultrasonogram revealed weakened myocardial contractility. Arterial blood pressure was normal. Suspecting late-onset sepsis, empirical antibiotic therapy was used. The laboratory tests showed thrombocytopenia (platelets: 40000/μL), low levels of inflammatory markers (CRP ≤5 mg/L, procalcitonin – 0.93 ng/mL), coagulation disorder (activated partial thromboplastin time [APTT] – 230.9 seconds, prothrombin time [PT] – 40.1 seconds, international normalised ratio [INR] – 4.3) and severe metabolic acidosis. The child was intubated and mechanically ventilated. Symptoms of haemorrhagic diathesis were observed. The weakened contractility of the heart muscle, low cardiac output and massive bleeding from the lungs and stomach persisted. The treatment included infusion of dopamine and milrinone, followed by norepinephrine, to manage the insufficient systemic perfusion. Acid–base balance was restored, and clotting disorders were treated with active agent VII, cryoprecipitate and platelet products. Red cell concentrates were used because of bleeding. Despite all these treatments, the child’s condition deteriorated. Approximately 6 hours after the first symptoms, cardiac arrest occurred. In spite of resuscitation measures, the child died. No bacterial growth was found in the post-mortem material (blood). The presence of Coxsackie B3 virus genetic material was confirmed by virological tests (in myocardial biopsy, faeces, pericardial sac fluid and throat) using reverse transcription polymerase chain reaction (RT-PCR) (PB-03/ Neuro) and genome sequencing. No tests were performed on the boy’s mother.

## Case 2 – myocarditis with severe arrhythmia and cardiac insufficiency

A male neonate was transferred on the day of his birth to the Neonatal Intensive Care Unit of Department of Paediatrics in Cracow from the regional hospital because of prematurity and respiratory and circulatory insufficiency. The child was delivered at 29 weeks’ gestation to a gravida 1 mother by Caesarean section, with body weight of 1900 g. The child was assessed using the Apgar scale at 1/3/5/10 minutes, getting 3/5/5/6 points, respectively. Caesarean section was performed due to severe vomiting of the mother who had polyhydramnios and arrhythmia with heart failure symptoms (ascites) in the foetus. The foetus had been examined 3 weeks earlier and no pathology had been found. One month before the birth, the father had been diagnosed with hand, foot and mouth disease. After the birth, the child was intubated and mechanically ventilated. Because of supraventricular tachycardia (≥225/minute) with narrow QRS complex and symptoms of circulatory failure, treatment with adenosine and continuous infusion of vasopressor amines was applied.

The newborn was admitted in a critical condition with intubation, was mechanically ventilated with 100% oxygen and was poorly responsive, with his saturation being volatile. Physical examination showed petechiae, haemorrhagic formations. Audible crepitations over the pulmonary fields and silent systolic murmur above the heart could be heard. Basic laboratory tests showed elevated levels of troponin T (254.7 ng/L; normal: ≤14 ng/L) and signs of anaemia (haemoglobin [Hb] 10.1 g/dL, hematocrit Ht 31.7%), levels of inflammatory markers were low (CRP≤5 mg/L). The bacteriological cultures on the day of admission (blood, stool and aspirate) were negative. The ultrasound examination showed ascites, hepatomegaly and hepatic vein dilatation, significantly reduced systolic function and cardiac dilatation. Lung ultrasound showed ‘white lungs’ syndrome. Empirical antibiotic therapy was started with ampicillin plus amikacin and aciclovir. Surfactant was administered.

The boy had experienced recurrent episodes of paroxysmal supraventricular tachycardia up to the third day of hospitalisation, which required multiple administrations of adenosine and amiodarone. Further, treatment with vasopressor amines (dopamine, milrinone and noradrenaline) continued due to circulatory failure. The boy required mechanical ventilation until the seventh day. The patient’s blood was tested by RT-PCR (PB-03/NEURO) for enterovirus infection, which turned out to be negative. However, due to a positive history of viral infection in the family, the parents were also tested with RT-PCR (PB-03/ NEURO) for Coxsackie virus infection, which was confirmed in the stool of the child’s mother .

The boy was discharged home in the 11th week of his life in a good general condition. No arrhythmias have been observed since the second week of his life. No myocardial dysfunction was found in the control echocardiogram.

## Case 3 – meningitis with seizures

A female newborn with an uncomplicated birth history was admitted to the ward on the 19th day of her life because of the following conditions: fever (up to 38°C); three episodes of body stretching, backward-bending of the head and pushing out of the tongue; and simultaneous convergent strabismus. The family history was as follows: the father of the child had developed a fever a few days earlier and the older siblings had experienced a mild respiratory infection. Upon admission to the ward, the child was in a fair condition and responsive. The Moro reflex was increased, occurring many times during the first hours after admission without an obvious trigger.

The CRP and PCT were negative (CRP≤5 mg/L, PCT 0.12 ng/ mL); complete blood count showed leucocytosis (19770/μL), with lymphocytes dominating in a smear (51.5%). Troponin T level was slightly above normal (50.1 ng/mL). The patient underwent lumbar puncture, which showed cytosis (1910 cells/μL), of which 1890 (98.9%) were mononuclear cells. Biochemical parameters of the fluid were normal.

In a cerebral ultrasound, a small cyst in the left choroid plexus was found. Initially, broad-spectrum antibiotic therapy with cephotaxim and ampicillin was introduced.

During the hospitalisation, the child was apathetic, poorly responsive, and eventually did not wake up for feeding. Diagnostic tests showed that the inflammatory markers were low, leucocytes decreased to 11910/μL. The ultrasound revealed thalamostriate vasculopathy. A lumbar puncture was repeated the next day (fifth day of hospitalisation). The cytosis in CSF was 68 cells/μL, with prevalence of mononuclear cells. On the same day, a positive result for presence of enteroviruses in stool samples was indicated by the National Institute of Hygiene. In the following days, a gradual improvement of the child’s general condition was observed; the girl became vital, woke up for feeding and started to eat eagerly.

The girl was discharged home in good general condition on Day 11.

## Discussion

Subjective examination, despite the ongoing progress in medicine, is still an essential part of the diagnostic process. In the case of young infants and newborn with enterovirus infections, it is invaluable for making a proper differential diagnosis and providing adequate treatment. It saves time and allows for prediction of complications.

Enterovirus infections are common worldwide and are one of the most common aetiological factors of virus infections in the paediatric population. These infections occur all year round in tropical climates and are seasonal in temperate climates, attaining the peak in the warm season [1, 2]. However, in our work, we do not observe such a trend (Table 1), as confirmed by the literature Enterovirus infections are diagnosed and treated regardless of the season as they are found all year round. Transmission occurs through the faecal– oral route, through inhalation, via placenta and directly from the mother during labour and probably during breastfeeding [3]. Enteroviruses can also be transmitted by touching various objects and by medical personnel. The incubation period is 3–6 days. While the infection is the most contagious in the first 2 weeks, the virus is excreted through oral and nasal excretions from 1 to 3 weeks [1] and is found in the stool for much longer periods of time, ranging from several weeks to several months (usually up to 3 months) from the onset of infection. Ease of transmission, long period of infectivity and numerous existing serotypes are the causes of a plethora of enterovirus infections. However, non-specific symptoms and their mild nature cause doctors to be complacent in naming or seeking the aetiology of those infections.

However, physicians working with children and pregnant women should be aware that there is a risk of severe course of enterovirus infection for some people, i.e. newborns and children <3 months of age [3]. The most common aetiological factors in this group are coxsackie B virus, echovirus and parechovirus [4]. The symptoms in newborns usually develop during the first 2 weeks of life. The course of infection can vary greatly from mild self-limiting to fulminant, life-threatening forms. The most common mild symptoms include fever, aversion to food, apathy or hyperesthesia, rash, gastrointestinal symptoms (such as diarrhoea) or respiratory tract-related symptoms, i.e. coughing and dyspnoea. Severe infection is manifested by systemic enteroviral sepsis with multi-organ failure, meningitis, myocarditis or fulminant hepatitis and coagulation disorders [3, 5]. Risk factors for severe course in newborns include early onset of the infection, prematurity, male gender, multi-organ failure (myocarditis and liver failure in particular), and infection caused by echovirus 11, parechovirus 3 and coxsackie B 1-5 [3, 4].

In most cases, the source of the infection is the sick mother or sick family members. Nearly 60% of mothers [6] whose children developed the enterovirus-induced disease in the perinatal period had an infection with fever in the week before delivery. Therefore, in the case of enteroviral infections, family history is crucial and helps in making an initial diagnosis when the symptoms are non-specific. The subjective examination should not be omitted at any stage – it should be done by gynaecologist-obstetrician, neonatologist and paediatrician. It should not be omitted or marginalised due to the severe condition of a child, as a minor viral infection of a mother or any household member may be fatal for the newborn.

Additionally, it is believed that the most important factor determining the course of the disease in newborns is the presence of neutralising antibodies (IgG) transmitted during pregnancy from the mother. For this reason, it is recommended (if possible) to delay the delivery by at least 5–7 days from the onset of symptoms in the mother [3]. However, in order to make such a decision, it is crucial to take the complete history and to be aware of the great benefits that such management may bring to the newborn.

Patient 1 with enteroviral sepsis had all the risk factors for a severe course of disease; the mother developed a fever on the day of delivery. Due to premature placenta detachment, delivery could not be delayed. Initially, the newborn was stable. The respiratory support that he needed was related to prematurity, and the patient did not show any signs of viral infection. The inflammatory markers remained normal. Available sources report that nearly a third of all cases present a two-phase course of the disease, in which the first phase is characterised by the asymptomatic period lasting 1–7 days, followed by symptoms of a severe infection [6]. Exactly on the seventh day of the child’s life, his condition suddenly deteriorated and fulminant viral sepsis developed, resulting in the child’s death. Only the history of perpetual fever in the mother, cooperation of medical teams and more detailed virological diagnostics could have confirmed the enterovirus infection, and the diagnostic and therapeutic management could have been modified based on the results. Moreover, it would have been possible to prepare for possible scenarios, make an early diagnosis and consider the use of intravenous infusion of Igs.

The family history was positive in all presented patients. The boy who had an arrhythmia (Case 2) also had a father with hand, foot and mouth disease, and further, the presence of enteroviruses was confirmed in the stool of his mother. Because of this information, we could count on favourable prognosis despite the extremely difficult course during the struggle for the patient’s life.

Patient 3 had a positive history of infections, as did her father, who was feverish a few days before the onset of symptoms in our patient. Further, in most cases, the family history was positive among the remaining children analysed in our study and most often, it concerned siblings of the child who presented symptoms of mild respiratory or gastrointestinal infection.

Of course, in children <3 months of age, fever with unknown origin regardless of the medical history is a reason to thoroughly assess it. According to worldwide-accepted guidelines, every newborn with a fever without source should be hospitalised and the decision concerning children < 3 months of age should be made only following medical evaluation. In these patients, the exclusion of serious bacterial infection, including sepsis, meningitis, urinary tract infections, pneumonia, osteoarthritis, bacterial enteritis and so on, is required. No laboratory test can be used as a screening method to determine the need for hospitalisation – subjective and objective examination is essential here. However, the tests may indicate bacterial or viral aetiology, especially if performed several times. Among our patient population, despite the clinical picture corresponding to serious bacterial diseases, laboratory tests (CRP, PCT and morphology) did not deviate significantly from the standards.

An important part of the differential diagnosis of fever in children <3 months of age is also the lumbar puncture, performed to exclude infections of the central nervous system. During the procedure, especially when a viral aetiology is suspected (based on history, physical examination and low inflammatory markers), it is important to secure an additional sample of cerebrospinal fluid (CSF) for virological examination. Each ward where newborns are hospitalised should know which laboratory performs such tests, what volume of CSF should be secured and under what conditions the sample should be stored and transported. It is worth noting that a virological examination of CSF should also be performed when results of the general examination of CSF are normal [8]. In one of our patients who presented with hyperesthesia, aversion to food and fever, the presence of enteroviral genetic material in the CSF was detected, despite the absence of abnormalities in the general CSF examination. Similar cases have also been described in the literature [3]. Children with meningitis presented hyperesthesia and anxiety; in one case, seizures were observed. Moreover, the majority of these patients also had gastrointestinal symptoms such as abdominal flatulence and diarrhoea, which were reported to be the dominant symptoms by the mothers. As such, it is evident that subjective and physical examination, rather than isolated laboratory tests, should play the most important role in the diagnostic process.

Infections in children <3 months of age often manifest as myocarditis[8]. The clinical picture is dominated by feeding difficulties, apathy and tachycardia at rest, sometimes cardiac arrhythmias. In this case, heart rhythm monitoring and imaging becomes the basis for diagnosis. Troponin T is also an important diagnostic marker. Currently, despite the widespread use of this test in adults, no reference standards are available in the paediatric population. Several consecutive measurements of troponin levels have the greatest predictive value to determine the trend.

Such symptoms were also observed among our patients with myocarditis, although not all of them occurred at the same time. Patient No. 10 (Table 1) presented, in addition to the fever, a persistent tachycardia at rest, no appetite and drops in saturation while feeding. Laboratory tests showed only increasing troponin levels in the first days of hospitalisation, which then gradually decreased. The remaining parameters did not deviate from the reference standards. Echocardiography showed no abnormalities.

Significantly more severe course of the disease with rhythm disturbances occurred in Patient 2. It was most probably linked to the first symptoms that started in the prenatal period and to the premature birth.

Currently, there is no causal treatment against enterovirus infections [1,9]. Studies conducted on Pleconaril and Pocapavir did not allow introduction of these drugs for common use [3,7]. Similarly, there are no clear indications for the use of Ig infusions. They can be considered as a treatment option for children born prematurely. Preterm infants who are deprived of the transplacental IgG transfer that would normally happen during the last weeks of pregnancy may constitute a special group in which the supply of Igs may be beneficial, similarly to newborns whose mothers have developed an infection in the period preceding delivery. Another option for the treatment of perinatal infections could be compatible plasma transfusion from a member of the family, who had been already cured from the infection[5]. This requires further research to determine the procedures for this specific group.

Further, the lack of a properly collected history and virological screening causes excessive use of combined and prolonged antibiotic therapy. After confirmation of the enterovirus infection in our patients, the antibiotics were discontinued and the only treatment our patients received was symptomatic. It is worth noting, however, that the waiting time for the enterovirus test results is often too long.

The prognosis of enteroviral infections, even a serious one, is generally good. Even enteroviral meningitis is described as the mildest case of meningitis. Therefore, proper identification of such infections has an important prognostic value.

## Conclusion

Enterovirus infections are common infections that should be taken into consideration during the diferential diagnosis of a febrile newborn or infant. Despite the reported seasonality, infections may also occur in other periods. One should keep in mind the key role of an epidemiological inquiry. It is important to pay attention to the family history as early as possible in the obstetrics and neonatal wards because this is where the cascade of undesirable events often begins. An early suspicion of a viral infection in the mother and securing of appropriate test materials may significantly speed up diagnosis in the newborn and help implement appropriate treatment.
